# Methane-Derived Carbon in the Benthic Food Web in Stream Impoundments

**DOI:** 10.1371/journal.pone.0111392

**Published:** 2014-10-31

**Authors:** John Gichimu Mbaka, Celia Somlai, Denis Köpfer, Andreas Maeck, Andreas Lorke, Ralf B. Schäfer

**Affiliations:** Institute for Environmental Sciences, University of Koblenz-Landau, Landau, Rhineland-Palatinate State, Germany; Scottish Association for Marine Science, United Kingdom

## Abstract

Methane gas (CH_4_) has been identified as an important alternative source of carbon and energy in some freshwater food webs. CH_4_ is oxidized by methane oxidizing bacteria (MOB), and subsequently utilized by chironomid larvae, which may exhibit low δ^13^C values. This has been shown for chironomid larvae collected from lakes, streams and backwater pools. However, the relationship between CH_4_ concentrations and δ^13^C values of chironomid larvae for in-stream impoundments is unknown. CH_4_ concentrations were measured in eleven in-stream impoundments located in the Queich River catchment area, South-western Germany. Furthermore, the δ^13^C values of two subfamilies of chironomid larvae (i.e. Chironomini and Tanypodinae) were determined and correlated with CH_4_ concentrations. Chironomini larvae had lower mean δ^13^C values (−29.2 to −25.5 ‰), than Tanypodinae larvae (−26.9 to −25.3 ‰). No significant relationships were established between CH_4_ concentrations and δ^13^C values of chironomids (p>0.05). Mean δ^13^C values of chironomid larvae (mean: −26.8‰, range: −29.2‰ to −25.3‰) were similar to those of sedimentary organic matter (SOM) (mean: −28.4‰, range: −29.3‰ to −27.1‰) and tree leaf litter (mean: −29.8 ‰, range: −30.5‰ to −29.1‰). We suggest that CH_4_ concentration has limited influence on the benthic food web in stream impoundments.

## Introduction

Allochthonous and autochthonous plant organic matter are major sources of carbon and energy for freshwater ecosystems [1]. Recent studies have revealed that also methane, which can be produced by microbial degradation of organic matter under anoxic conditions, can significantly contribute to the carbon budget of freshwater ecosystems [2]. Part of this gas is released to the atmosphere, where it contributes to the pool of green house gases [3], or is oxidized by methane oxidizing bacteria (MOB) [4]. The biogenic methane in MOB can contribute to the biomass of chironomid larvae [5]. Chironomids are one of the most dominant invertebrate groups in the soft sediments in freshwater ecosystems and their larvae feed mainly on algae or allochthonous organic material and associated microroganisms [6]. CH_4_ derived organic carbon may constitute a crucial source of carbon and energy for chironomids, in comparison to other aquatic invertebrates, because their burrowing habit creates and exposes them to oxyclines at the sediment-water interface, where MOB density and CH_4_ oxidation rates are usually high [7]–[10]. For example, chironomid larvae collected from some lakes were sustained (up to 70%) by CH_4_ derived carbon [11].

These quantitative estimates are based on the stable carbon isotope signature (δ^13^C) of CH_4_ which is highly depleted due to carbon isotopic fractionation related to methanogenesis [12]. Additionally, MOB that oxidize CH_4_ are usually characterized by further depletion in δ^13^C [13]. Therefore, organisms that consume MOB have lower δ^13^C values (typically <−40‰; [14]), in comparison to organisms that feed on plant organic matter (−32 to −21 ‰; [15]). Bunn & Boon [16] determined the δ^13^C values of invertebrates in backwater pools and found Chironominae larvae to have δ^13^C values (<−35‰) that were lower than for particulate organic matter (−29 to −25 ‰). Kiyashko et al. [18] and Jones & Grey [19] also reported lower (−64 to −55 ‰) δ^13^C values for some chironomid larvae than for particulate organic matter in lakes. The observed differences between δ^13^C values of chironomid larvae and potential food resources led to the conclusion that the chironomid larvae might have fed on MOB, which has very low δ^13^C values. The carbon isotope composition of consumers (e.g. insects) is determined by their diet and usually potrays an enrichment by about 1 ‰, even though the δ^13^C can deviate from −3 ‰ to +3 ‰ [20]. Given that methane is isotopically very distinct, stable carbon isotopes are particularly useful for tracing methane derived carbon [17].

Although most existing studies on the importance of CH_4_ derived carbon in freshwater food webs mainly focused on lakes [2], a wide array of anoxic habitats with high potential for CH_4_ production also exist in rivers and streams [21]–[23]. Particularly, impoundments increases the residence time of water, promotes accumulation of organic matter and sediment, and have been identified as hot spots of CH_4_ emissions [24]. Maeck et al. [24] measured CH_4_ concentrations in riverine and impoundment reaches and found sediment accumulation in dams to be the main source of CH_4_ emissions. Guérin et al. [25] reported an increase in CH_4_ emissions at the downstream sides of impoundments as a result of release of water enriched with CH_4_.

In shallow aquatic systems such as rice paddies and small lakes, CH_4_ has been shown to be an important source of energy in the benthic food webs [26]–[28]. In spite of the high abundance of small in-stream ponds in smaller streams [29], the relationship between CH_4_ concentrations and stable carbon isotope ratios (δ^13^C) of chironomid larvae in such systems has not been examined. Globally, there exist millions of small impoundments (height <15 m; [30]). Within this study, we assessed (i) CH_4_ concentrations in stream and pore-water and (ii) the relationship between CH_4_ concentrations and δ^13^C values of chironomid larvae in impoundments located in the Queich River catchment area, South-western Germany. We hypothesized that CH_4_ would have a significant influence on the δ^13^C of chironomid larvae.

## Methods

### Ethics statement

This study was conducted in the Queich River catchment area (see coordinates below) and was not conducted in an area requiring research permit (e.g. national park) or private land. This study did not involve endangered or protected species.

### Study area and sites

The study was conducted in the Queich River catchment area, Rhineland-Palatinate State, South-western Germany. The Queich River (length: 52 km) originates from the Palatinate forest (49^o^10′6^o^N 7^o^50′48^o^E) and flows (mean discharge: 1.31 m^3^ s^−1^; www.geoportal-wasser.rlp.de) through the upper Rhine Valley to its confluence with the Rhine River in Germersheim (49^o^13′39^o^N 8^o^23′4^o^E). The catchment (area: 271 km^2^) is primarily covered by sandstone and is between 100 m and 673 m above sea level. The Rhineland-Palatinate region has dry climate conditions in summer.

**Figure 1:**
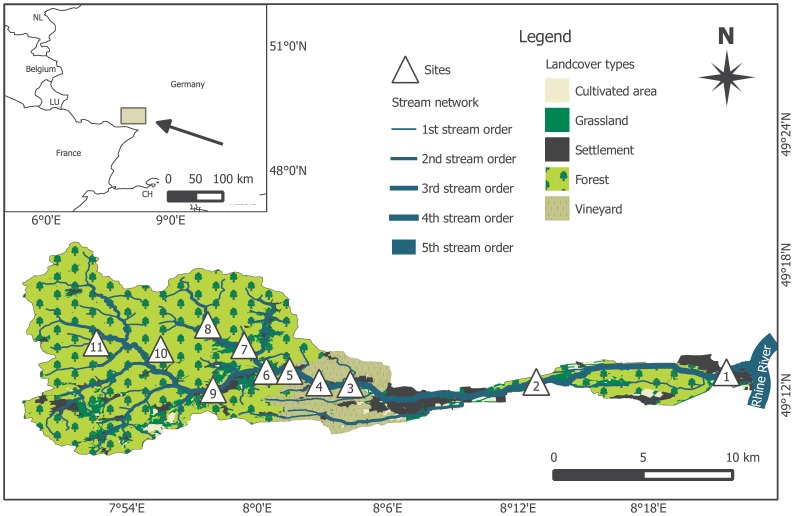
Locations of the study impoundments in the Queich River catchment area.

Typical for most stream networks in central Europe, 67 small in-stream impoundments (www.geoportal-wasser.rlp.de) have been constructed on the main stem and tributaries of the Queich River, South-western Germany, for various purposes such as hydropower generation and flood control. Here, we selected eleven study sites (e.g. Figure S1 in File S1) located from the downstream to the upstream reaches of the Queich River catchment area (Figure 1, Table S1 in File S1). The study sites were located at five different streams. The sampled impoundments were approximately 0.5–2.0 metres deep and had water bypasses that transported water to the downstream reaches.

### Water chemistry and physical characteristics

Field measurements and sampling were conducted between 9^th^ and 24^th^ June, 2013. Data collection was done between 9 a.m. and 4 p.m. Electrical conductivity, temperature, dissolved oxygen concentration and pH of stream water were measured *in situ* with a WTW Multi 340i/SET (Wissenschaftlich Werkstätten GmbH, Weilheim, Germany). Average water depth was computed from three measurements taken on a transect across the river channel and current speed was estimated by timing a float over a distance of 5 metres [31]. Water discharge was calculated from velocity, width and depth [31]. Water residence time was calculated as follows:



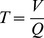
(i)where: *T* is the water residence time, *V* is the volume of water stored in the impoundment, and *Q* is the water discharge [32]. Nitrate and phosphate concentrations in stream water were determined in the laboratory using Macherey-Nagel viscolor kits (Macherey-Nagel, Düren, Germany).

### CH_4_ concentrations

Concentrations of dissolved CH_4_ in stream and pore-water were measured at the impoundments. Water samples were collected from each study impoundment using 20 mL serum bottles. The samples for stream water CH_4_ analysis were collected by filling water to the sample bottles from the bottom to top, and overflowing the sample bottles several times over. Three bottles were completely filled with water at each sampling site and several drops (250 µL) of mercuric chloride were added to each bottle as preservative [33]. The bottles were capped and sealed and transported to the laboratory. A headspace was prepared by replacing 10% of the bottle (i.e. 2 mL) with nitrogen gas. To generate the headspace, each sample bottle was held upside down and a 20 gauge needle was inserted through the septum. Then 2 mL nitrogen gas was added to each bottle using a syringe, while the replaced water sample escaped through the needle. The samples were manually shaken, for 1 minute, to equilibrate the gas between the headspace and the water [34]. The samples were analyzed using a CH_4_ analyser (Los Gatos Research Inc., Mountain view, CA, U.S.A.). A closed loop was created between the gas inlet and outlet of the analyser. A gas tight syringe was then used to inject 0.5 mL gas sample into the closed loop. The CH_4_ concentrations were averaged over 30 seconds before and after gas injection. Concentration of the injected gas was computed as:
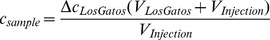
(ii)where: *c_sample_* is the mol fraction of the sampled gas in parts per million, *Δc_LosGatos_* is the change in mole fraction before and after gas sample injection, *V_LosGatos_* = 92.5 mL and *V_Injection_* = 0.5 mL.

Sediment samples for pore-water CH_4_ analysis were obtained at the impoundments, where fine sediment accumulated. Pore-water CH_4_ concentrations were assessed from sediment cores (1 core per site). Cores were taken at location of soft sediment using a piston corer and analyzed for porosity, carbon:nitrogen (C:N) ratio, total organic carbon (TOC) content and pore-water CH_4_ concentration. Cut-off syringes (3 mL) were used to extract sediment sub-samples which were immediately placed into crimp capped 20 mL vials containing 3 mL of 2.5% NaCl solution for conservation of the CH_4_. Pore-water was sampled in the cores from the homogenized upper (0–10 cm) sediment layer, where chironomid larvae are found [35]. The pore-water CH_4_ samples were measured as described for water samples. For C:N ratio, TOC and porosity, three sediment sub-samples were extracted from the cores (0–10 cm) and placed into glass tubes before analysis in the laboratory.

### Chironomid sampling and processing

Chironomid samples were collected from the deepest point in each impoundment using an Ekman grab sampler (Hydro-Bios, Kiel, Germany). Sediments were sieved by passing them through two metal sieves (mesh size: 500 µm and 2 mm). Materials such as stones and large pieces of organic matter (>5 cm) were removed and chironomid larvae were picked from a sorting tray using forceps and placed into 500 mL sample bottles containing river water. In some sites only few chironomids (<5 chironomids) were found. The chironomid samples were transported to the laboratory and transferred to sample bottles containing clean tap water for 24 hours to allow gut clearance. Faecal materials were periodically removed to prevent ingestion by chironomids [36]. Chironomids were sorted by tribe and subfamily [37]–[40] and size (instar) [41], [42] under a dissecting microscope (magnification: x 40-100). Chironomids of the same tribe, subfamily and size were pooled to obtain sufficient mass (0.5–1 mg) for isotope analysis [43]. Sorting of chironomids by size was done to detect the potential effect of body size on the isotopic signal as demonstrated by Grey et al. [44]. In most cases the limited number of specimens excluded replicate analyses. Therefore, we collected individuals from a site with a high abundance of chironomids to exemplarily determine the δ^13^C variability from 9 replicates. Second instar larvae were discarded as they were too small for identification and their mass was insufficient for isotope analysis. Before isotope analysis, chironomids were placed into glass tubes, oven dried at 60°C for 24 hours and subsequently stored in a desiccator.

### Stable isotope, TOC and C:N analyses

Three replicate sediment samples, from each site were analysed, for δ^13^C of sedimentary organic matter (SOM), TOC and C:N ratios. They were rinsed with a 2.5% HCL solution for four hours to remove carbonates [45], rinsed three times with demineralised water, oven dried at 60°C for 24 hours and ground using a mortar and pestle. Leaves, for analysis of δ^13^C of potential allochthonous food resources, were collected from trees near the impoundments, washed with demineralised water, rinsed, oven dried and ground before analysis.

Sediment samples for C:N ratios and TOC were weighed into tin cups (15–20 g) and analysed using a Vario Microcube elemental analyser (Elementar Analysensysteme, Hanau, Germany). The chironomid, SOM and leaf litter samples for stable isotope analysis were also weighed (approximately 0.5–1.0 mg for chironomids and 5–20 mg for SOM and leaf litter) into tin cups before their combustion in an isotope ratio mass spectrometer (ThermoScientific, Bremen, Germany). Stable isotope ratios were expressed in per mille (‰).

### Statistical analysis

Relations between variables were tested using Spearman's rank correlation test [46]. Comparisons of CH_4_ concentrations in pore and stream water, and between δ^13^C values of chironomids, and leaf litter and SOM were done using paired t-test. A value of *p*<0.05 was considered as statistically significant. Homogeneity of variances was examined with Bartlett's test and data were square root transformed to improve normality. Statistical analyses were done using the R statistical package [47] and all data are provided in File S1.

## Results

### Water chemistry and physical characteristics

Water residence time varied from 0.5 to 6.0 minutes. Nutrient concentrations in stream water ranged from 3.5 to 5.0 mg NO_3_ L^−1^, and from 0.1 to 0.3 mg PO_4_ L^−1^. Water temperature ranged from 10.2 to 18.6°C, whereas electrical conductivity, dissolved oxygen concentrations and water discharge ranged from 61 to 380 µS cm^−1^, 8.5 to 11.9 mg L^−1^ and 0.2 to 7.8 m^3^ s^−1^ (Table S1 in File S1).

### Methane gas concentrations

Average values of dissolved CH_4_ concentrations in stream water ranged from 0.07 µmol L^−1^ at Site 6 to 0.7 µmol L^−1^ at Site 10 (Table 1). Pore-water CH_4_ concentrations ranged from 0.3 µmol L^−1^ at Site 7 to 1657.5 µmol L^−1^ at Site 9, and were statistically significantly (t-value  = 3.1, *p* = 0.005) higher than the stream water CH_4_ concentrations. Some pore-water CH_4_ measurements at sites 3 and 6 differed greatly from the other measurements, either due to disturbance during sampling or gas leakage during analysis, and were therefore taken to be unreliable and excluded from further analysis.

**Table 1:** Average CH_4_ concentrations (µmol L^−1^) and bulk sediment characteristics (TOC, C:N ratio, porosity and δ^13^C) of samples from the studied impoundments (in parentheses are standard errors, ±SE (when applicable).

Name	Code	Pore-water CH_4_	Dissolved CH_4_	TOC (%)	C:N ratio	Porosity	δ^13^C (‰)
Germersheim	1	27.8 (4.6)	0.2 (0.006)	2.8 (0.1)	17.6 (0.8)	0.6 (0.02)	−28.7 (0.01)
Fuchsbach	2	658.1 (244.4)	0.4 (0.004)	3.1 (0.1)	23.2 (0.9)	0.7 (0.02)	−28.2 (0.3)
Godramstein	3	142.5	0.3 (0.04)	0.2 (0.02)	12.0 (1.0)	0.4 (0.02)	−27.1 (0.02)
Siebeldingen	4	1499.6 (784.6)	0.1 (0.06)	2.8 (0.03)	17.1 (0.6)	0.7 (0.003)	−28.8 (0.04)
Albersweiler Pfalz	5	199.6 (8.2)	0.1 (0.004)	0.6 (0.04)	16.1 (1.2)	0.5 (0.02)	−28.9 (0.2)
Rosenfeldt Mill	6	263.0	0.07 (0.001)	1.8 (0.04)	16.4 (0.6)	0.5 (0.03)	−28.5 (0.1)
Eußerbach	7	0.3 (0.01)	0.3 (0.01)	0.3 (0.03)	15.2 (1.4)	0.4 (0.03)	−29.3 (0.2)
Eisbach	8	45.2 (8.7)	0.2 (0.03)	1.6 (0.2)	14.7 (0.6)	0.6 (0.02)	−28.6 (0.1)
Annweiler Am Trifels	9	1657.5 (128.9)	0.1 (0.002)	2.4 (0.2)	19.5 (1.2)	0.6 (0.01)	−28.7 (0.1)
Langenbächel	10	4.6 (2.2)	0.7 (0.07)	0.9 (0.1)	17.9 (1.5)	0.6 (0.02)	−28.9 (0.04)
Modenbach	11	105.9 (18.3)	0.3 (0.01)	0.5 (0.02)	16.1 (0.6)	0.3 (0.01)	−27.3 (0.1)

### δ^13^C of SOM and leaf litter, C:N ratios and TOC

δ^13^C values of SOM ranged from −29.3‰ at site 7 to −27.1‰ at site 3 (Table 1), whereas δ^13^C values of leaf litter ranged from −30.5 ‰ to −29.1‰. C:N ratios of sediment and the TOC ranged from 12.0 at site 3 to 23.2 at site 2, and from 0.2% at site 3 to 3.1% at site 2, respectively (Table 1).

### δ^13^C of chironomids

**Figure 2:**
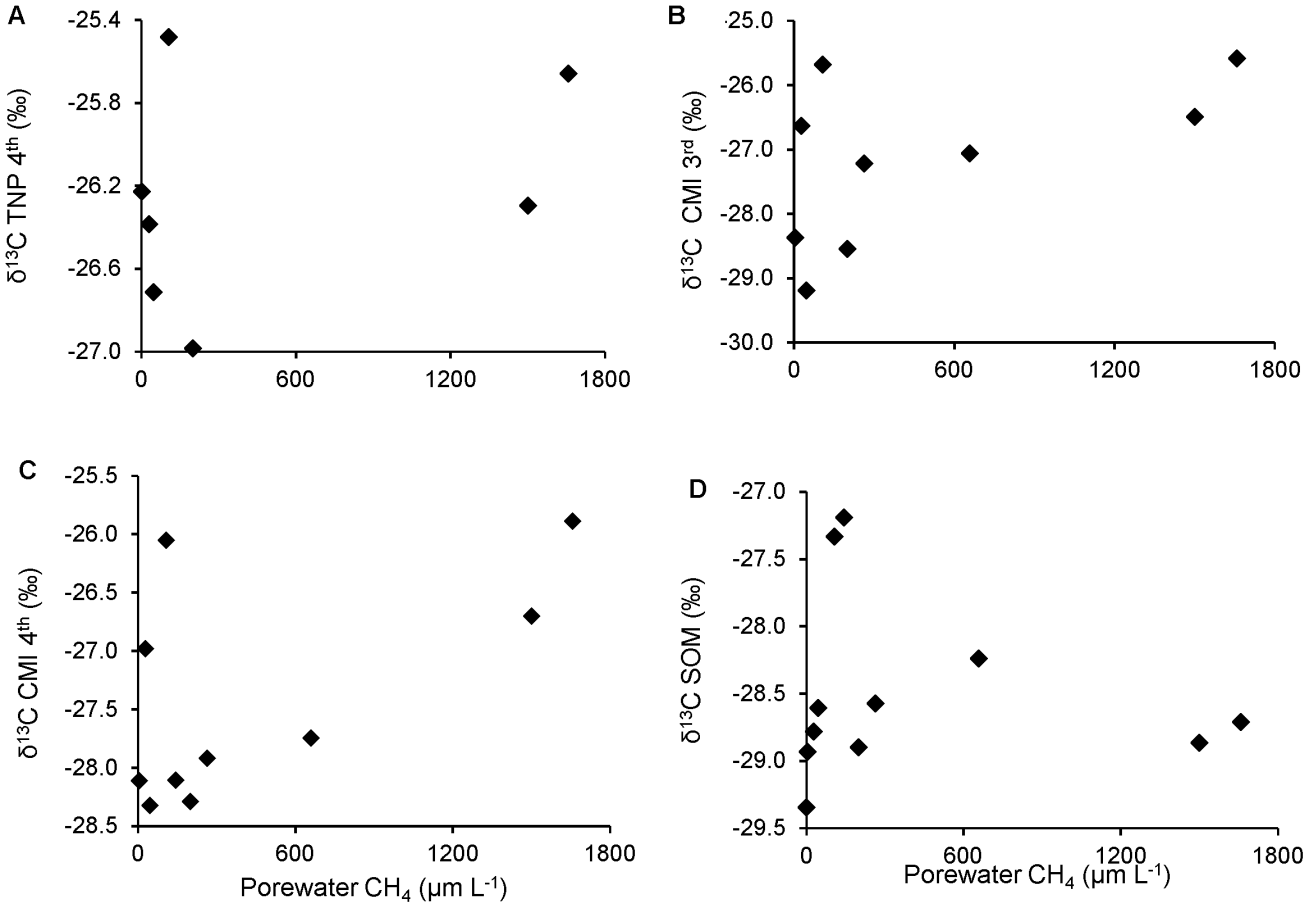
Relationships between pore-water CH_4_ concentrations and δ^13^C values. TNP 4^th^, CMI 3^rd^, CMI 4^th^ and SOM stands for Tanypodinae fourth generation, Chironomini third and fourth generation, and sedimentary organic matter, respectively.

The chironomids were identified as Chironomini, Tanypodinae and *Chironomus* sp. *Chironomus* sp. were only found at site 10 and were not analyzed because they did not provide an adequate pooled mass for isotope analysis. The lowest δ^13^C value, −29.2 ‰, was measured in a third instar Chironomini larvae collected from site 8, whereas the highest δ^13^C values, −25.3 ‰, were measured in third instar Tanypodinae larvae collected from Sites 9 and 11 (Table S2 in File S1). Generally, the highest mean δ^13^C values were measured in third (−25.3±0.01‰) and fourth (−26.3±0.14 ‰) instar Tanypodinae, whereas slightly lower mean δ^13^C values were measured in fourth (−27.2±0.16 ‰) and third (−26.9±0.25 ‰) instar Chironomini. δ^13^C values did not differ significantly between third and fourth instar Chironomini larvae (t-value  = 1.4, *p* = 0.17). The analysis of 9 replicates of fourth instar Chironomini larvae from site 4 had a standard deviation as low as 0.19 ‰ for δ^13^C (Table S2 in File S1). No significant correlations were observed between CH_4_ concentrations and δ^13^C values of chironomids (Figure 2, Table 2). However, δ^13^C values of chironomid larvae were significantly correlated to those of the SOM and to each other within site (*p*<0.05) (Table 2). Mean δ^13^C of chironomid larvae (−26.8 ‰) was more similar to that of the SOM (−28.4 ‰) than leaf litter (−29.8 ‰) and there were significant differences (*p*<0.05) between δ^13^C values of chironomids, and SOM and leaf litter.

**Table 2:** Correlations (*rho*-values) between δ^13^C values, C:N ratio of bulk sediment, water residence time (min) and CH_4_ concentrations (^*^ *p*<0.05).

	δ^13^C_SOM_	Dissolved methane	Pore-water methane	CMI 4^th^	TNP 4^th^	Water residence time	CMI 3^rd^
C:N_SOM_	−0.16	−0.28	0.35	−0.46	−0.15	−0.55	−0.29
δ^13^C_SOM_		−0.80*	0.30	0.66*	0.92*	−0.28	0.76*
Dissolved CH_4_			−0.78*	−0.11	−0.57	0.22	−0.28
Pore-water CH_4_				−0.50	−0.05	0.21	−0.35
CMI 4^th^					0.87*	−0.41	0.97*
TNP 4^th^						−0.53	0.95*
Water residencetime							−0.55

δ^13^C_SOM_, δ^13^C of bulk sediment; CMI 4^th^, fourth instar Chironomini; CMI 3^rd^, third instar Chironomini; TNP 4^th^, fourth instar Tanypodinae.

## Discussion

### Methane gas concentrations

CH_4_ concentrations measured in this study are comparable to those measured in other aquatic ecosystems [48]–[50]. Pore-water CH_4_ concentrations showed a highly variable pattern among the impoundments (Table 1). These differences can be attributed to heterogeneity in the distribution of sedimentary organic materials within the impoundments. Sanders et al. [51] found pore-water CH_4_ concentrations to be influenced by sediment heterogeneity. The authors reported that enhanced retention of sediment by macrophytes (*Ranunculus penicillatus*) increased pore-water CH_4_ production in streams. Significantly higher mean CH_4_ concentrations were recorded in pore than stream water. This can be explained by the fact that CH_4_ is usually produced in sediments, where anoxic conditions are likely to develop, whereas the stream water is rather well oxygenated (Table S1 in File S1) [21].

### Relationship between δ^13^C of chironomids and methane gas

Few studies examined CH_4_ as a source of carbon and energy for stream invertebrates (e.g. [52]). In these studies, mean δ^13^C values of invertebrates supported by CH_4_ derived carbon were lower (<−40 ‰) than those of potential photoautotrophic food resources, indicating ingestion and assimilation of MOB, which had oxidized isotopically light CH_4_. This was also demonstrated for lake invertebrates [7], [43], [53], where significant negative relationships between CH_4_ concentrations and δ^13^C values of some invertebates indicated ingestion and assimilation of MOB.

Mean δ^13^C of chironomid larvae ranged from −27.2 (fourth instar Chironomini) to −25.3 ‰ (fourth instar Tanypodinae). The mean δ^13^C values of potential food resources ranged from −28.4 (SOM) to −29.8 ‰ (leaf litter) and were significantly correlated to those of the chironomids. The similarity of δ^13^C of Chironomini larvae and SOM can be attributed to the fact that Chironomini larvae are either filterers or gathering-collectors, feeding on fine particulate organic matter in aquatic systems [54]. In comparison to Chironomini, Tanypodinae utilize different types of food (e.g. detritus, oligochaetes, diatoms; [55]) and their δ^13^C values may be difficult to interpret when compared with the other chironomids. The differences between the δ^13^C values of chironomids, SOM and leaf litter were within the reported range (±3 ‰) of δ^13^C values for consumers and their food resources [20].

In the current study, no significant relationships were established between CH_4_ concentrations in stream and pore-water and δ^13^C values of chironomid larvae. In the UK, Trimmer et al. [52] found river water to have higher mean (0.16 µmol L^−1^) CH_4_ concentrations than pore-water (0.07 µmol L^−1^). Highly depleted mean δ^13^C values (<−42 ‰) of Trichoptera larvae (*Agapetus fuscipes*), relative to those of potential food sources (−38 ‰), were only measured in areas with low pore-water CH_4_ concentrations. In comparison to Trimmer et al., we recorded higher mean CH_4_ concentrations in stream and pore-water and the mean δ^13^C values of chironomid larvae were not low. The type of food consumed by chironomids and presence of MOB influence the δ^13^C values of chironomid larvae. The short water residence times and the shallow nature of the studied impoundments could have enhanced water turn over rates, mixing of the entire water column and supply of organic matter or periphyton into the benthic zone. Thus, in the case of chironomid larvae feeding on sedimenting organic matter, we would anticipate their δ^13^C to be similar to that of their food resources. Mixing of the water column may also increase oxygen concentration and CH_4_ oxidation rates, and reduce CH_4_ production and the biomass of MOB available to chironomids [9]. For example, Eller et al. [9] recorded two fold higher MOB density in the anoxic waters (0.1 mg L^−1^ O_2_) of a stratified lake than in the well mixed and oxygenated waters (9 mg L^−1^ O_2_) of a polymictic lake. Additionally, the contribution of MOB to chironomid larvae biomass was higher in the anoxic than oxygenated waters. Jones et al. [11] found that the contribution of MOB to the chironomid larvae biomass was highest at sites with low dissolved oxygen content (2–4 mg L^−1^). Within the above mentioned studies, the isotopic values of chironomids were more similar to those of the SOM, when they were collected from sediments overlaid by well oxygenated waters. Use of MOB was particularly pronounced under hypoxia or post mixing following on from stratification.

δ^13^C values of chironomid larvae (mean  =  ∼−27 ‰; −29 to −25 ‰) were similar to those of SOM (mean  =  ∼−28 ‰; −29 to −27 ‰) and leaf litter (mean  =  ∼−29 ‰; −30 to −29 ‰). Additionally, δ^13^C values of chironomid larvae were significantly correlated to each other. Given that the δ^13^C values varied little between consumers and their food sources [56], the chironomid larvae collected from the sampled impoundments most likely obtained their carbon through ingestion and assimilation of SOM or allochthonous leaf litter. Other studies using stable carbon isotope analysis also demonstrated SOM and allochthonous plant organic matter as significant sources of carbon and energy for freshwater invertebrates [57]. The C:N ratio can be used to determine the source of organic matter in aquatic ecosystems because autochthonous organic matter generally has lower C:N ratios (e.g. algae: 4–10; [58]) than allochthonous organic matter. The measured C:N ratios (12.0–23.2) of sediments indicated elevated proportions of allochthonous organic matter.

Although the δ^13^C values of chironomid larvae did not indicate utilization of methane derived carbon, other invertebrates could have used it as a source of energy. For example, Kohzu et al. [59] found coleopterans collected from backwater pools to have lower mean (−40 to −67 ‰) δ^13^C values than the other invertebrates (e.g. chironomids; −36 ‰), suggesting increased utilization of methane derived carbon. In summary, this study reveals that methane derived carbon did not contribute substantially to chironomid larval biomass in small impoundments, rather that allochthonous organic matter was the main source of energy. Future studies assessing the role of methane derived carbon in stream impoundments should include MOB community characterization, CH_4_ oxidation rates and fluxes, and δ^13^C values of other invertebrates.

## Supporting Information

File S1 **Supporting figure and tables.** Figure S1, Example of a weir that impounded the studied rivers. The white arrow shows the direction of water movement. Table S1, Location and environmental characteristics recorded from the studied impoundments. Table S2, δ^13^C values (‰) of chironomid larvae from the studied impoundments. For each of the impoundments, between 1 and 9 replicates (*n*) were made from pooled samples.
(DOC)
