# Supplementary material for: Methane-Derived Carbon in the Benthic Food Web in Stream Impoundments
Source: PLoS One. 2014 Oct 31;9(10):e111392. doi: 10.1371/journal.pone.0111392 (PMC4216073; doi:10.1371/journal.pone.0111392)
Supplement: File S1 — Supporting figure and tables. Figure S1, Example of a weir that impounded the studied rivers. The white arrow shows the direction of water movement. Table S1, Location and environmental characteristics recorded from the studied impoundments. Table S2, δ13C values (‰) of chironomid larvae from the studied impoundments. For each of the impoundments, between 1 and 9 replicates (n) were made from pooled samples. (DOC) [file pone.0111392.s001.doc]

**Supporting information for the paper:** Methane-derived carbon in the benthic food web in stream impoundments

By: John Gichimu Mbaka, Celia Somlai, Denis Köpfer, Andreas Maeck, Andreas Lorke, Ralf B. Schäfer

**This file contains one figure and two tables:**

**Figure S1** Page 2

**Table S1** Page 3

**Table S2** Page 4


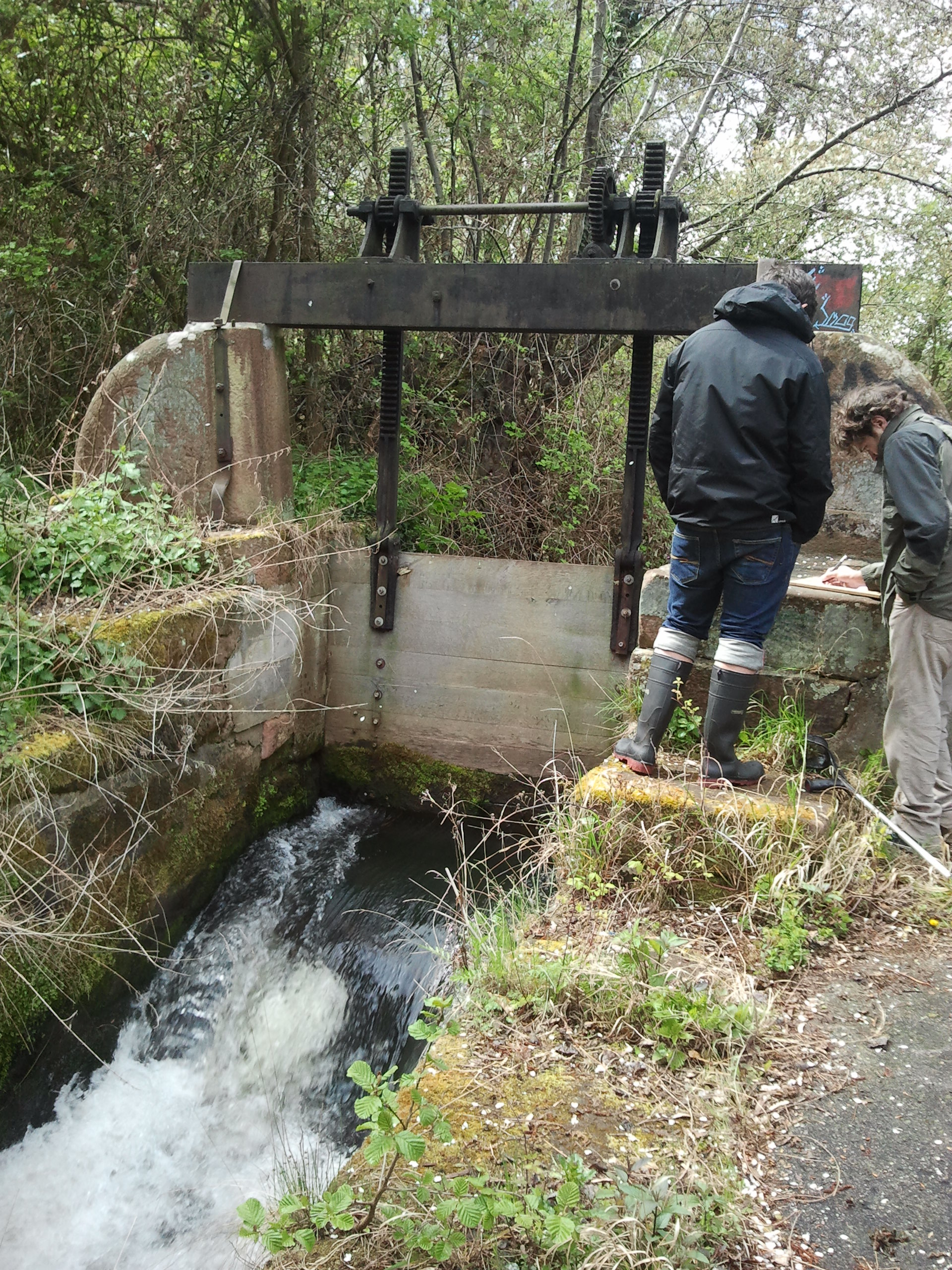


| Impoundment  name | Code | Coordinates | Mean  Depth  (cm) | pH | Water discharge  (m^3^ s^-1^) | O2  (mg  L^-1^) | Water residence time (min) | Temperature  (^o^C) | Conductivity  (µS cm^-1^) | NO^-3^  (mg L^-1^) | PO^-4^  (mg L^-1^) | Main  land  use |
| --- | --- | --- | --- | --- | --- | --- | --- | --- | --- | --- | --- | --- |
| Germersheim | 1 | N49^o^21´77´´,  E8^o^35´97.7´´ | 65 | 7.7 | 7.80 | 9.4 | 0.10 | 15.3 | 329 | 5.0 | 0.25 | Urban |
| Fuchsbach | 2 | N49^o^21´03´´,  E8^o^21´39.5´´ | 37 | 6.9 | 0.51 | 8.8 | 0.63 | 18.6 | 376 | 5.0 | 0.30 | Urban |
| Godramstein | 3 | N49^o^20´86´´,  E8^o^07´14.4´´ | 74 | 7.5 | 2.60 | 9.6 | 0.50 | 13.0 | 234 | 3.5 | 0.20 | Urban |
| Siebeldingen | 4 | N49^o^20´98´´,  E8^o^04´74.6´´ | 162 | 7.7 | 0.97 | 11.9 | 5.00 | 13.8 | 229 | 5.0 | 0.30 | Urban |
| Albersweiler  pfalz | 5 | N49^o^20´86´´,  E8^o^07´14.4´´ | 50 | 7.4 | 1.48 | 11.6 | 0.66 | 13.1 | 191 | 4.5 | 0.20 | Urban |
| Rosenfeldt  Mill | 6 | N49^o^21´87´´,  E8^o^00´69.7´´ | 82 | 7.5 | 2.28 | 11.3 | 0.41 | 15.5 | 267 | 5.0 | 0.20 | Urban |
| Eußerbach | 7 | N49^o^25´41´´,  E7^o^96´21.5´´ | 78 | 7.2 | 0.15 | 11.9 | 0.83 | 10.2 | 81 | 5.0 | 0.10 | Forest |
| Eisbach | 8 | N49^o^23´84´´,  E7^o^99´00.1´´ | 35 | 7.8 | nd | 10.2 | nd | 17.4 | 380 | 5.0 | 0.30 | Forest |
| Annweiler AmTrifels | 9 | N49^o^20´45´´,  E7^o^96´61.5´´ | 65 | 7.2 | 1.62 | 10.7 | 0.66 | 14.0 | 234 | 5.0 | 0.30 | Urban |
| Langenbächel | 10 | N49^o^23´55´´,  E7^o^92´6´´ | 75 | 7.0 | nd | 8.5 | nd | 15.4 | 89 | 3.5 | 0.15 | Forest |
| Modenbach | 11 | N49^o^24´04´´,  E7^o^87´67.6´´ | 127 | 6.4 | 1.64 | 11.7 | 5.83 | 13.1 | 61 | 5.0 | 0.15 | Forest |

*nd – not detected

| Impoundment name | Code | Species and instar  (by size) | *n* | δ^13^C (‰) | | | |
| --- | --- | --- | --- | --- | --- | --- | --- |
|  |  |  |  | Mean | S.D. | Min | Max |
| Germersheim | 1 | Chironomini 4^th^ instar | 3 | ‒26.9 | 0.48 | ‒26.4 | ‒27.3 |
|  |  | Chironomini 3^rd^ instar | 3 | ‒26.6 | 0.16 | ‒26.4 | ‒26.7 |
|  |  | Tanypodinae 4^th^ instar | 1 | ‒26.3 |  |  |  |
| Fuchsbach | 2 | Chironomini 4^th^ instar | 1 | ‒27.7 |  |  |  |
|  |  | Chironomini 3^rd^ instar | 1 | ‒27.1 |  |  |  |
| Godramstein | 3 | Chironomini 4^th^ instar | 1 | ‒28.1 |  |  |  |
| Siebeldingen | 4 | Chironomini 4^th^ instar | 9 | ‒26.7 | 0.19 | ‒26.4 | ‒27.1 |
|  |  | Chironomini 3^rd^ instar | 3 | ‒26.4 | 0.04 | ‒26.4 | ‒26.5 |
|  |  | Tanypodinae 4^th^ instar | 4 | ‒26.2 | 0.11 | ‒26.1 | ‒26.4 |
| Albersweiler | 5 | Chironomini 4^th^ instar | 4 | ‒28.2 | 0.19 | ‒28.1 | ‒28.5 |
|  |  | Chironomini 3^rd^ instar | 3 | ‒28.5 | 0.22 | ‒28.2 | ‒28.7 |
|  |  | Tanypodinae 4^th^ instar | 3 | ‒26.9 | 0.02 | ‒26.9 | ‒26.9 |
| Rosenfeldt | 6 | Chironomini 4^th^ instar | 3 | ‒27.9 | 0.04 | ‒27.8 | ‒27.9 |
|  |  | Chironomini 3^rd^ instar | 3 | ‒27.2 | 0.34 | ‒26.9 | ‒27.5 |
| Eußerbach | 7 | Tanypodinae 4^th^ instar | 1 | ‒26.2 |  |  |  |
| Eisbach | 8 | Tanypodinae 4^th^ instar | 1 | ‒26.7 |  |  |  |
|  |  | Chironomini 3^rd^ instar | 1 | ‒29.2 |  |  |  |
| Annweiler | 9 | Chironomini 4^th^ instar | 3 | ‒25.8 | 0.04 | ‒25.8 | ‒25.9 |
|  |  | Chironomini 3^rd^ instar | 3 | ‒25.5 | 0.16 | ‒25.3 | ‒25.6 |
|  |  | Tanypodinae 4^th^ instar | 1 | ‒25.6 |  |  |  |
|  |  | Tanypodinae 3^rd^ instar | 1 | ‒25.3 |  |  |  |
| Längenbachel | 10 | Chironomini 4^th^ instar | 1 | –28.1 |  |  |  |
| Modenbach | 11 | Chironomini 4^th^ instar | 3 | ‒26.1 | 0.14 | ‒25.9 | ‒26.2 |
|  |  | Chironomini 3^rd^ instar | 3 | ‒25.6 | 0.06 | ‒25.6 | –25.7 |
|  |  | Tanypodinae 4^th^ instar | 3 | ‒25.4 | 0.16 | ‒25.3 | ‒25.5 |
|  |  | Tanypodinae 3^rd^ instar | 1 | ‒25.3 |  |  |  |
